# A sensitivity analysis framework for the treatment effect measure used in the meta-analysis of comparative binary data from randomised controlled trials

**DOI:** 10.1002/sim.5591

**Published:** 2012-09-02

**Authors:** Dan Jackson, Rose Baker, Jack Bowden

**Affiliations:** a MRC Biostatistics UnitCambridge, U.K.; b University of SalfordSalford, U.K.

**Keywords:** binary data, meta-analysis, sensitivity analysis

## Abstract

The process of undertaking a meta-analysis involves a sequence of decisions, one of which is deciding which measure of treatment effect to use. In particular, for comparative binary data from randomised controlled trials, a wide variety of measures are available such as the odds ratio and the risk difference. It is often of interest to know whether important conclusions would have been substantively different if an alternative measure had been used. Here we develop a new type of sensitivity analysis that incorporates standard measures of treatment effect. Thus, rather than examining the implications of a variety of measures in an *ad hoc* manner, we can simultaneously examine an entire family of possibilities, including the odds ratio, the arcsine difference and the risk difference. Copyright © 2012 John Wiley & Sons, Ltd.

## 1. Introduction

Performing a meta-analysis involves making a number of important decisions. The Cochrane Handbook [1], Section 9.7, describes several of these and advocates sensitivity analysis as a suitable approach for evaluating their implications. In particular, under the heading of ‘Analysis methods’, the Cochrane Handbook asks ‘For dichotomous outcomes, should odds ratios, risk ratios or risk differences be used?’ Deeks and Altman [2] and Sinclair and Bracken [3] provide good accounts of the issues involved in choosing a suitable measure and, in particular, distinguish between relative (e.g. the odds ratio) and absolute (e.g. the risk difference) measures of treatment effect.

Other outcome measures for comparative binary data are also available, including the recently proposed arcsine difference[4]. This measure was proposed for the analysis of trials with zero or small counts, and we regard this as an important measure to incorporate into our methods. For example, odds ratios or risk differences may have been identified, *a priori*, as the outcome measure to be used in analysis. If, however, some or many studies are subsequently found with zero counts, then the implications of using the arcsine difference may be of particular interest. The choice between a relative or an absolute measure may be especially crucial when the event is rare, so we suggest that a sensitivity analysis may be particularly important in such instances.

The implications of the choice of outcome measure was also investigated by Deeks [5], who examines which type of measures for comparative binary data appears to be the most consistent. We agree with Deeks that the choice of outcome measure should not be determined by the ‘best fitting’ model but rather that this should be guided by empirical evidence and clinical debate. Deeks finds that the risk difference is a less consistent measure than the relative measures he investigated, and interest in quantifying the impact of heterogeneity has subsequently increased. We therefore consider the now very popular *I*^2^ statistic[6] resulting from the choice of outcome measure to be an important quantity to explore.

We assume that the data are from randomised controlled trials, so that all the usual measures of treatment effect are appropriate and may easily be calculated. We also assume that the meta-analytic data are in the common form where in each study there are two treatment groups and we have counts for the number of participants who experience and do not experience the event of interest. These data can be presented in the form of a series of two by two tables.

Although sensitivity analyses may be criticised on the grounds that they do not provide a single answer, in addition to the Cochrane Handbook's recommendation, several authors have suggested using sensitivity analyses in the context of meta-analysis. For example, Copas and Shi [7] and Bowden *et al*. [8] suggest using them when assessing publication bias. The term ‘sensitivity analysis’ covers a wide range of strategies, but the approach adopted here is to introduce a sensitivity parameter that describes the type of outcome measure used.

The rest of the paper is set out as follows. In Section 2, we introduce our generalised outcome measure, and we show how the standard measures are special cases of this. In Section 3, we describe our proposed procedure for a sensitivity analysis, and in Section 4, we apply this to some examples. We conclude with a discussion in Section 5.

## 2. A more general measure of treatment effect for comparative binary data

All of the standard measures of treatment effect for comparative binary data from randomised controlled trials are based upon a suitable transformation of the estimated probabilities of an event. Let 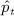 and 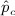 denote the estimated probability of an event in the treatment and control groups in a particular study, respectively. Then, in general, the estimated treatment effect in this study is given by


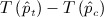
(1)

where *T*(*θ*) denotes the transformation corresponding to the treatment effect used. For example, *T*(*θ*) = logit(*θ*) provides the log-odds ratio, and trivially *T*(*θ*) = *θ* provides the risk difference.

We desire a more general measure of treatment effect, where this measure depends on a single sensitivity parameter *λ*. We also require that this measure incorporates standard measures as special cases, so that these will be embedded into our sensitivity analysis that follows.

We motivate our more general measure using variance-stabilising transformations because these are natural transformations that aid normality and so provide a good starting point. A variance-stabilising transformation uses the delta method to obtain a transformed variable for which the variance is approximately constant. For proportions that obey the binomial distribution, the variance-stabilising transformation is the arcsine transformation[9], which may be written as


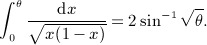
(2)

This result can be shown by substituting *x* = sin ^2^*y*, and it seems natural to generalise this type of integral slightly to


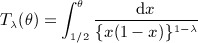
(3)

where *λ* takes values in the interval [0,1]; *λ* > 1 is also possible, but we will consider values in this interval. Hence, we generalise (2) by allowing the use of alternative powers in the integral. Equation (3) provides a suitable transformation to use in (1) when defining a treatment effect because it is strictly increasing in *θ*. By taking 1 / 2 as the lower limit in the integration, *θ* = 1 / 2 is transformed to zero, which aids interpretation. Note that (3) transforms all *θ* in the interval [0,1]: if *θ* < 1 / 2, then we define


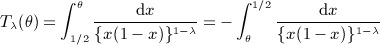


as usual. Finally, by changing variables *z* = 1 − *x*, we obtain *T*_*λ*_(1 − *θ*) = − *T*_*λ*_(*θ*) so that the transformation possesses good symmetry properties.

A related idea is the transformation suggested by Aranda-Ordaz [10] in his Equation (1). When differentiating his *T*_*λ*_(*x*) with respect to *x*, we obtain


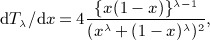


which shows that this alternative transformation is based on a similar type of integral. However, we prefer our transformation (3) because this incorporates the standard meta-analytic measures of treatment effect more directly.

### 2.1. Some important special cases that the general measure incorporates

The estimate of treatment effect from a particular study, using the transformation (3) in (1), is given by


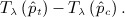
(4)

This incorporates three important measures of treatment effect as special cases. It is straightforward to show that *T*_0_(*θ*) = logit(*θ*), and so we obtain the log-odds ratio as the measure of the treatment effect if we take *λ* = 0 in (6). We obtain the linear transformation when *λ* = 1. More specifically, when *λ* = 1, (3) becomes *θ* − 1 / 2. Hence, we obtain the risk difference as the measure of the treatment effect in (6) if we take *λ* = 1. Finally, from (2) we obtain *twice* the arcsine difference if we take *λ* = 1 / 2.

Although not popular in meta-analysis, the probit transformation (Collet [11], Section 3.5.2) is another commonly used link function in generalised linear modelling for binary outcome data. This transformation was also considered by Aranda-Ordaz [10], who found that his transformation could approximate this well. We can see numerically that *T*_0.3_(*θ*) ≍ Φ^− 1^(*θ*), provided *θ* is not very close to zero or one, where Φ^− 1^( ⋅ ) denotes the inverse of the standard normal cumulative distribution function. Hence, we can interpret *λ* = 0.3 as approximately providing an outcome measure that we will refer to as the probit difference. Other link functions are also available for binary data, and future work may explore how these may also be approximated using our transformation. We suggest an extension of our general measure, which incorporates the log relative risk, in the discussion.

### 2.2. Modelling the study outcomes using the general measure of treatment effect

We use linear (Taylor series) approximations to justify the use of a normal approximation for the treatment effect so that standard meta-analysis methods may be used. We return to the possibility of using exact binomial distributions, rather than normal approximations, in the discussion. Upon using linear approximations for 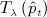 and 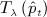, (6) becomes


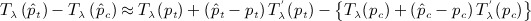
(5)

where 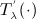 denotes the derivative of 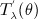 with respect to *θ*. From (3), we can evaluate 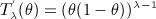. We assume that the studies are large enough so that normal approximations may be used for both 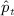 and 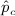, which also we assume are independent. Then (7) shows that the estimated treatment effect is (approximately) a linear combination of two independent normal random variables and so is also normally distributed.

We make the standard assumptions that 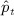 and 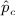 are unbiased estimators of the probability of an event in each treatment group, that is, 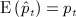 and 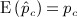. This is, however, only approximately true if ‘corrections’ are made to avoid zero counts as explained subsequently. We also make use of the standard result that the variances of these proportions are 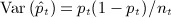 and 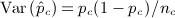, where *n*_*t*_ and *n*_*c*_ are the numbers of subjects in the two groups. By using these results and taking the expectation of (7), we obtain


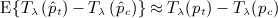
(6)

and similarly, upon further replacing unknown parameters with their estimates


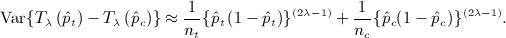
(7)

From (8), the estimate (6) is an approximately unbiased estimate of the corresponding study specific underlying effect, and from (9) we obtain the within-study variance. This method of obtaining within-study variances is commonly referred to as the delta method. Most standard within-study variance formulae in meta-analysis are derived in this way, but this method is not without its problems[12].

When *λ* = 0 and *λ* = 1, we obtain the standard formulae for the variance of an empirical log-odds ratio and risk difference, respectively. When *λ* = 1 / 2, we obtain 1 / *n*_*t*_ + 1 / *n*_*c*_ as the variance of twice the arcsine difference. This asymptotic variance is less than the conservative variance proposed by Rücker *et al*. [4], who also propose an analytical alternative. We, however, adopt the asymptotic variance here because this allows the results from the arcsine difference to be accommodated in our sensitivity analysis in a straightforward manner, but we return to this issue in the discussion.

Hence, we can apply any standard meta-analysis model and method that uses data in the form of estimates and within-study variances when using our measure. In particular, this includes the random effects model[13], for example, by assuming that the estimates of treatment effect are distributed as 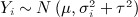, where *μ* is the average effect and *τ*^2^ is the between-study variance.

### 2.3. Evaluating the general measure of treatment effect

The only practical difficulty when obtaining within-study variances from (9) is due to zero counts, which result in either zero or infinite within-study variances, depending on the value of *λ*. Hence, we suggest using some type of ‘correction’, such as the common practice of adding halves[14], in situations where zero counts are encountered. This also avoids the difficulties associated with estimating the log-odds ratio ( *λ* = 0) when there are zero counts.

Evaluating the treatment effect itself for an arbitrary *λ* is apparently more difficult, however, because (6) requires two integrals of the form (3) to be evaluated. Although these integrals are very easily computed analytically for the special cases considered so far, this is not the case more generally. However, in the current computational climate, this presents no real problems. For those with access to numerical integration routines, for example, the necessary integrals could be evaluated numerically. In the Appendix, we provide another method that does not require numerical integration but instead requires the use of standard statistical functions. The method described in the Appendix was used to produce all the results in this paper because it is faster.

### 2.4. Interpreting the general measure of treatment effect

Although our more general measure of treatment effect corresponds to some important special cases for particular values of *λ* and it possesses the good properties described earlier, it is less clear how to interpret it more generally. In this section, we provide a way to interpret the measure as (approximately) a linear combination of three established measures of treatment effect. Hence, all values of *λ* can be interpreted as corresponding to a weighted average of these measures. As explained subsequently, this also facilitates an understanding of the extent to which the general measure is relative or absolute, depending on the value of *λ*.

Barycentric Lagrange interpolation[15] will be used to aid interpretation. Here we use interpolation points at *λ* = 0,1 / 2,1, and require that our function takes the values logit (*θ*), 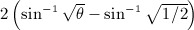 and (*θ* − 1 / 2) at these points. The interpolation (Reference [15], their Equations 3.2 and 4.2) gives


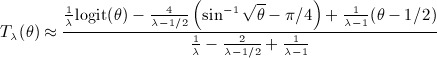


so that the treatment effect (6) is approximately


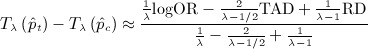
(8)

where ‘logOR’ denotes the log-odds ratio, ‘TAD’ denotes twice the arcsine difference and ‘RD’ denotes the risk difference. At *λ* = 0,1 / 2,1 we take the limiting results from (11) so that the generalised treatment effect is appropriately and exactly the conventional treatment effects as described in Section 2.1. From (11), we see that the treatment effect is approximately a weighted average of the three conventional measures for other values of *λ*, where the weights depend on *λ*. For example, we define the percentage weight of the log-odds ratio as


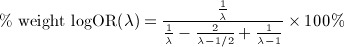


**Figure 1:**
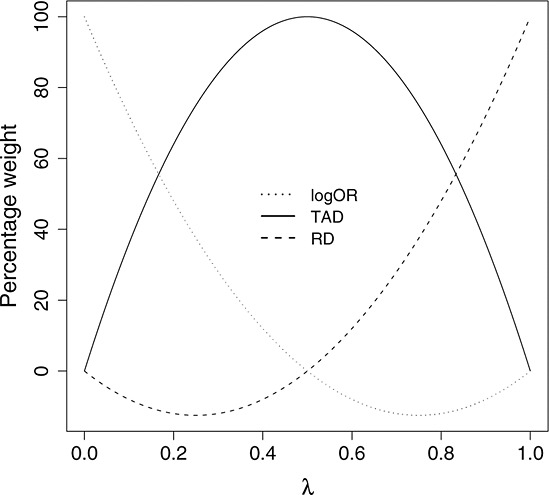
The approximate percentage weights that the three standard measures contribute to the generalised measure of treatment effect. The dotted line denotes the log-odds ratio, the dashed line denotes the risk difference and the solid line denotes twice the arcsine difference.

The approximate percentage weight that each measure contributes is plotted against *λ* in Figure 1. Because the weights are only approximate, we interpret the small negative weights in this figure as indicating that the corresponding measure contributes little or nothing to the generalised measure. Figure 1 then shows that the contribution of the log-odds ratio decreases as *λ* increases from zero and that the log-odds ratio contributes little or nothing when *λ ≥*1 / 2. Hence, the extent to which the generalised measure is a relative measure decreases to little or nothing over the interval [0,1 / 2]. Similarly, the risk difference contributes little or nothing until *λ* is as great as 1 / 2, and its contribution increases over the range [1 / 2,1]. Finally, (twice) the arcsine difference contributes to the measure for all *λ* ≠ 0,1; it contributes the most at *λ* = 1 / 2, and this decreases as *λ* moves away from 1 / 2 in either direction.

## 3. A sensitivity analysis

Now that we have established our generalised measure of treatment effect and shown how this corresponds to some established estimates of treatment effect, we are ready to develop our procedure for performing a sensitivity analysis for the treatment effect measure used. Our strategy is to allow *λ* to vary across the range [0,1]. As explained in Section 2, zero counts in the two by two tables present difficulties when using normal approximations; so halves, or some other value, are added as necessary to avoid this before analysing the data.

We then perform a meta-analysis using our generalised measure of treatment effect over a fine grid of *λ* in the interval [0,1]. We perform each of these meta-analyses using whichever variation of meta-analytic methodology we wish to apply, whether it be fixed or random effects, Frequentist or Bayesian and so forth. By plotting quantities of interest against *λ*, we can assess their sensitivity to the treatment effect measure used. Further, by recalling that *λ* = 0 corresponds to log-odds ratios, *λ* = 0.3 (approximately) corresponds to the probit difference, *λ* = 0.5 corresponds to *twice* the arcsine difference and *λ* = 1 corresponds to the risk difference, we can assess the sensitivity to the choice of these measures. Further, because the measure of treatment effect is continuous in *λ*, all quantities of interest will vary continuously with this, resulting in plots that are visually attractive as well as insightful.

## 4. Examples

We now illustrate our sensitivity analysis using two contrasting examples. In both instances, we apply random effects meta-analyses using the standard method of DerSimonian and Laird [16], and we present the results from this and the values of the *I*^2^ statistic in our sensitivity analyses. However, any variation of the standard meta-analytic methodology that takes binary data and creates a treatment effect that is modelled using a normal distribution could be almost as easily used instead. Furthermore, any other quantity of interest could also be investigated, such as the estimate of the between-study variance or a *p*-value.

### 4.1. Example 1: diuretics

**Figure 2:**
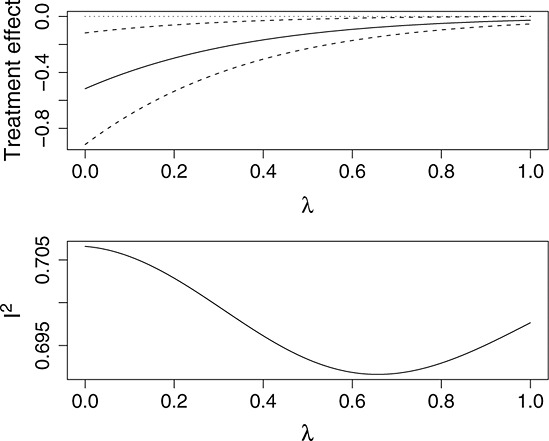
Sensitivity analysis for the diuretic data (example 1). The first plot shows the estimated effect and its 95% confidence interval, and the second plot shows the *I*^2^ statistic. The results for the log-odds ratio, the (approximate) probit difference, twice the arcsine difference and the risk difference are shown at *λ* = 0,0.3, 0.5 and 1, respectively.

Biggerstaff and Jackson [17] used this example, which involves the results from nine randomised controlled trials investigating the effects of diuretics on pre-eclampsia. The trials are generally quite large, and there are no zero counts. Biggerstaff and Jackson used the log-odds ratio as the outcome measure and focussed on inference concerning the between-study variance. Here we instead focus on the treatment effect and assess the sensitivity of the conclusions to the outcome measure used in a random effects meta-analysis. In particular, we are interested to see if other measures of treatment effect result in similar inferences to the log-odds ratio because this was used previously. A random effects meta-analysis using log-odds ratios (Figure 2; *λ* = 0) indicates a beneficial treatment effect because a negative log-odds ratio means that pre-eclampsia is less likely in the treatment group. Furthermore, this comfortably achieves statistical significance at the 5% level.

Figure 2 shows that a beneficial treatment effect is also inferred across almost the entire range of *λ*. *I*^2^ is very insensitive to the measure used and is quite large (around 0.7) for all analyses. The general impression from Figure 2 is that the inferences are qualitatively similar across all values of *λ* irrespective of the measure used and therefore the extent to which the outcome measure is relative or absolute. The only obvious potential concern is that the statistical significance at the 5% level becomes borderline if the risk difference is instead used as the outcome measure (Figure 2; *λ* = 1).

To summarise, Figure 2 reassures us that the inferences are not especially sensitive to the outcome measure used. The estimated treatment effect is statistically significant over a wide range of measures, which provides further weight to the belief that the treatment may be effective.

### 4.2. Example 2: off-pump surgery

**Figure 3:**
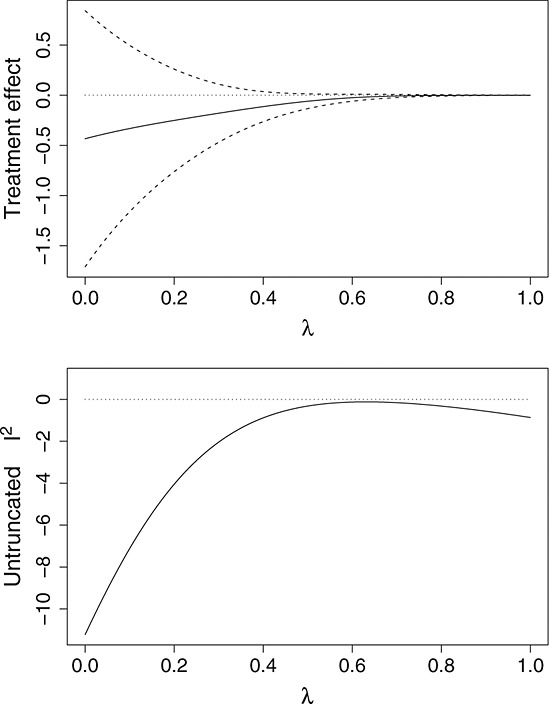
Sensitivity analysis for the off-pump surgery data (example 2). The first plot shows the estimated effect and its 95% confidence interval, and the second plot shows the untruncated *I*^2^ statistic. The results for the log-odds ratio, the (approximate) probit difference, twice the arcsine difference and the risk difference are shown at *λ* = 0,0.3,0.5 and 1, respectively.

We take this example from Rücker *et al*. [4], where the effect of off-pump surgery in coronary artery bypass grafting on postoperative stroke is examined in 21 studies. Rücker *et al*. used this example to illustrate the use of the arcsine measure, and hence there is particular interest in whether the inferences are sensitive to this choice of outcome measure. We use the random effects model, as in the previous example, but all estimates of the between-study variance (and hence the truncated *I*^2^ statistics) are zero. Hence, all random effects analyses collapse to fixed effect analyses, and the plot for the treatment effect parameter in Figure 3 also shows the results for fixed effect analyses. The event that a subject has a stroke is rare, and there are many zero counts (there are only 20 events in total), which probably explains why this occurs: with so few events, there is little potential for the data to provide evidence of between-study variation.

We replaced zeroes with 0.0001 times the sample size of the corresponding treatment group in order to avoid the problems associated with them. This also avoids imputing event rates that are greater than the positive observed rates and reflects the fact that the event of interest is very rare. As the arcsine measure does not require any corrections for zero counts, the results at *λ* = 0.5 are only approximately those of the arcsine measure where no correction is made, but we can still use these to assess the sensitivity to the outcome measure used.

This example is especially interesting because, although the estimated effect is statistically insignificant at the 5% level for all *λ*, the analysis using the log-odds ratio (Figure 3; *λ* = 0), a relative measure, permits quite a wide range of effects. The risk difference, however (Figure 3; *λ* = 1), is inferred to be small. This makes sense from the nature of the data, where there are moderately large studies with very rare events: we cannot be precise about the relative difference between the two treatment groups, but we do know the absolute difference is small. Figure 3 captures this perfectly; as the extent to which the measure is a relative measure drops over the interval [0,1 / 2], the confidence interval drastically shortens.

As mentioned earlier, the *truncated I*^2^ is zero over the entire range of *λ*, suggesting that this finding not sensitive to the type of treatment effect measure used, but in Figure 3 we plot the *untruncated* version of this statistic (*Q* − (*n* − 1)) / *Q*, where *Q* denotes Cochran's *Q* statistic and *n* is the number of studies. The untruncated *I*^2^ statistic is large and negative at *λ* = 0 but increases to almost zero over the interval [0,1 / 2], suggesting that the data are highly homogenous when relative measures are used, but statistical heterogeneity is almost apparent for more absolute measures. This observation nicely corroborates Deeks’ [5] finding that the risk difference is a less consistent measure than the relative measures he investigates.

To summarise, we can see from Figure 3 that inferences are qualitatively similar for different measures, but it is possible to make much more precise statements about absolute, rather than the relative, measures of treatment effect. More information is needed if precise statements about the relative effectiveness of the treatment are required. The arcsine measure therefore appears to be a good choice for the analysis of data where the event is rare. Not only does it possess good properties as explained by Rücker *et al*., but it can also be seen as a suitable compromise between the log-odds ratio and the risk difference in such instances.

## 5. Discussion

We have developed a sensitivity analysis in order to investigate the implications of using alternative measures of treatment effect for comparative binary data from randomised controlled trials. Our generalised measure of treatment effect includes several standard measures as special cases. It possesses the good symmetry property *T*_*λ*_(1 − *θ*) = − *T*_*λ*_(*θ*) and so does not include the relative risk *p*_*t*_ / *p*_*c*_. However, if the event is rare, as is the case in our second example, then the odds ratio, which is included, provides a good approximation to the relative risk of an event. More generally, if the meta-analyst is concerned about the implications of using the relative risk, of either harmful or beneficial outcomes, then we suggest that the sensitivity analysis be complemented by the results from these analyses. Because the relative risk is another commonly used outcome measure in meta-analysis, it may be implemented in many standard software packages, so this does not present much of a challenge in practice. Alternatively, we could generalise (3) slightly to


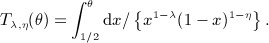


On taking *η* = 1 we obtain, to within a constant, the Box–Cox transformation. Thus, we can accommodate the logarithm of relative risk within our framework, with *η* = 1 and *λ* = 0, if one is willing to vary two parameters rather than one. This idea, which allows a wide range of transformations using two parameters, is perhaps akin to the use of fractional polynomials in regression modelling. Analogous approaches for alternative types of outcome, such as continuous or time to event data, await investigation.

Some commonly used methods for pooling data, such as the Mantel–Haenszel or Peto's method, are used for particular measures of treatment effect. Furthermore, there are variations when implementing the random effects model[18]. Thus, there may also be interest in the sensitivity of the inferences to the method used when pooling the studies’ results as well as the sensitivity to the outcome measure used. This provides another possibility to explore. A further issue is that, when using normal approximations, we require appropriate within-study variances. Rücker *et al*. [4] prefer their conservative and analytical within-study variances to the asymptotic within-study variance adopted here when the arcsine measure is used. When we use normal approximations, the sensitivity to the method for obtaining within-study variances may also be of interest.

We have used the very popular method proposed by DerSimonian and Laird, which is one of the simplest methods for pooling the study results. However, likelihood-based methods[19] are now much more computationally feasible than they were when this method was originally proposed. Methods for incorporating our generalised measure in a fully likelihood-based framework may form the subject of future work. One limitation of DerSimonian and Laird's method is that this uses normal approximations for the studies’ results, and this cannot be expected to provide a good approximation when the studies are small. Methods using the binomial distribution for binary meta-analytic data[20–23] have subsequently been proposed. In particular, Warn *et al*. [24] show how alternative measures for binary data that do not take values along the entire real line, like ours when *λ* > 0, can be modelled using a normal random effect upon adding the necessary constraints. Likelihood and/or Bayesian methods, using binomial distributions for the within-study distributions and a normally distributed random effect for our measure, are currently being developed. If the studies are small or the event is rare, we can expect this type of analysis to be an improvement on analyses that use the normal approximations that we applied here.

To summarise, we have shown how our generalised measure of treatment effect can be used to investigate the sensitivity of the results from a meta-analysis to the outcome measure used. *R* code is available from the first author to implement our proposed sensitivity analysis. This provides both the plots and the corresponding numerical output, which makes our method widely accessible to applied researchers.

## Appendix

In order to evaluate the treatment effect from (6), we need to evaluate two integrals of the form (3), so we require a method to evaluate

where

(9)

This is clearly the incomplete (symmetric) beta function. First, we set *x* − 1 / 2 = *z* to obtain

and next we set 1 − 4*z*^2^ = 1 / (1 + *t*^2^ / *ν*), where *ν* is arbitrary. Over the range (0,1 / 2), *z* increases from zero, and *t* increases monotonically with *z*. Hence, assuming *θ ≥*1 / 2, *z*(*t*) is a suitable function to use when performing integration by substitution, and so we proceed for now by assuming this. Then implicit differentiation gives

and after some manipulation, the integral becomes

Assuming that *λ* > 0 (if *λ* = 0, then the integral may be evaluated as the logit function using elementary methods), on choosing *ν* = 2*λ*, this becomes

This integrand is proportional to the distribution function of the *t*-distribution. Hence,

(10)

where *F* is the distribution function of the *t*-distribution with 2*λ* degrees of freedom.

As derived, (10) applies to *θ ≥*1 / 2. However, if *θ* < 1 / 2, then *T*_*λ*_(*θ*) may be evaluated by evaluating *T*_*λ*_(1 − *θ*) from (10) and then applying (9). However, evaluating *T*_*λ*_(*θ*) for *θ* < 1 / 2 in this way and using the symmetry around zero of the *t*-distribution shows that (10) applies to all 0 *≤ θ ≤*1.

Finally, it is interesting to note that this shows that, if *λ* > 0, then *T*_*λ*_(*θ*) is finite for all 0 *≤ θ ≤*1. If, however, *λ* = 0, then the integral fails to converge when *θ* = 0 or 1, which corresponds to an infinite log-odds ratio.
